# [5,10,15,20-Tetra­kis(4-meth­oxy­phen­yl)porphyrinato]zinc di­chloro­methane disolvate

**DOI:** 10.1107/S1600536813019338

**Published:** 2013-07-24

**Authors:** Sean McGill, Vladimir N. Nesterov, Stephanie L. Gould

**Affiliations:** a Department of Chemistry, Austin College, 900 North Grand, Sherman, TX 75090-4400, USA; b Department of Chemistry, University of North Texas, Denton, TX 76203-5017, USA

## Abstract

In the title compound, [Zn(C_48_H_36_N_4_O_4_)]·2CH_2_Cl_2_, the Zn^II^ ion lies on an inversion center and is coordinated in an almost ideal square-planar geometry. The asymmetric unit also contains one di­chloro­methane solvent mol­ecule. The unique meth­oxy-substituted benzene rings form dihedral angles of 59.38 (6) and 66.77 (6)° with the mean plane (r.m.s. deviation of fitted atoms = 0.0282 Å) of the atoms in the porphyrin core. The packing is characterized by close contacts between the Zn^II^ ion and two symmetry-related mol­ecules through the O atoms of a meth­oxy­phenyl group [Zn⋯O = 2.694 (2) Å], forming a two-dimensional network parallel to (100).

## Related literature

For related structures, see: Adilov & Thalladi (2007 ▶); Bhuyan & Sarkar (2011 ▶); Teo *et al.* (2003 ▶). For the synthesis, see: Adler *et al.* (1967 ▶). For van der Waals radii, see: Bondi (1964 ▶).
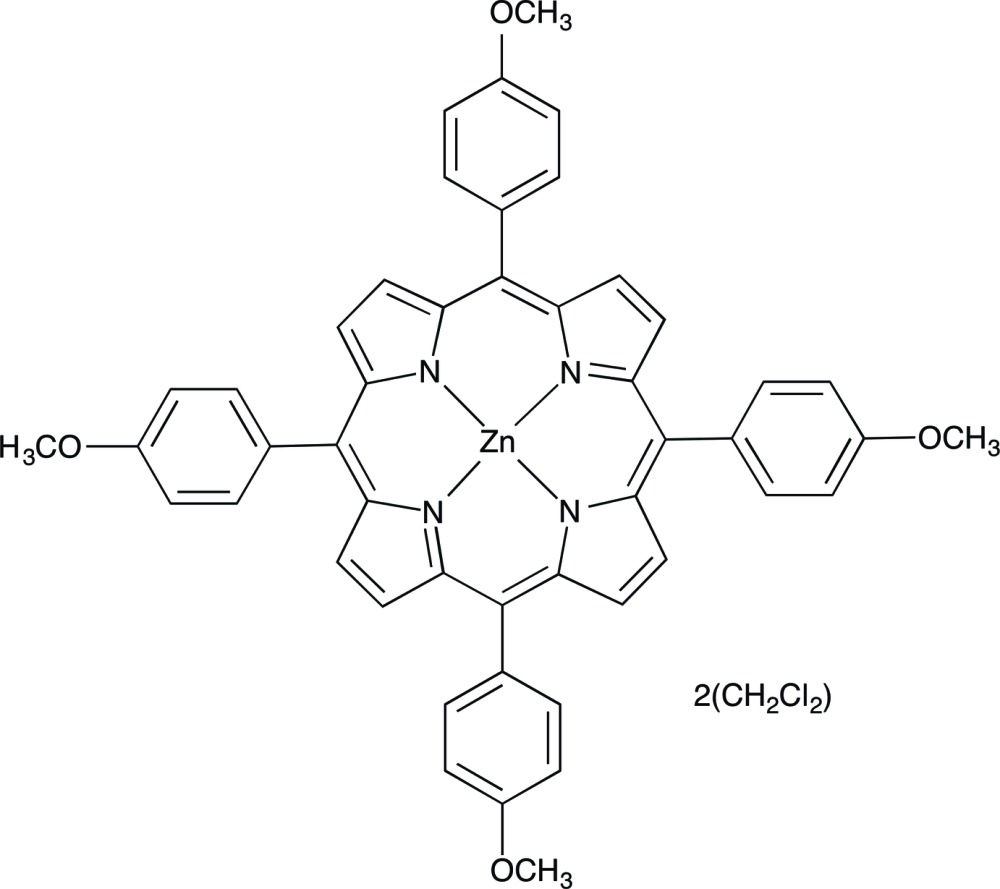


## Experimental

### 

#### Crystal data

[Zn(C_48_H_36_N_4_O_4_)]·2CH_2_Cl_2_
*M* *_r_* = 968.03
Monoclinic, 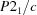

*a* = 11.4189 (9) Å
*b* = 10.6877 (9) Å
*c* = 18.3778 (15) Å
β = 106.022 (1)°
*V* = 2155.7 (3) Å^3^
*Z* = 2
Mo *K*α radiation
μ = 0.87 mm^−1^
*T* = 100 K
0.18 × 0.16 × 0.09 mm

#### Data collection

Bruker APEXII CCD diffractometer
Absorption correction: multi-scan (*SADABS*; Bruker, 2007 ▶) *T*_min_ = 0.861, *T*_max_ = 0.926
25964 measured reflections
4763 independent reflections
4022 reflections with *I* > 2σ(*I*)
*R*_int_ = 0.036

#### Refinement

*R*[*F* ^2^ > 2σ(*F* ^2^)] = 0.039
*wR*(*F* ^2^) = 0.121
*S* = 1.06
4763 reflections
288 parameters
H-atom parameters constrained
Δρ_max_ = 0.52 e Å^−3^
Δρ_min_ = −0.78 e Å^−3^

### 

Data collection: *APEX2* (Bruker, 2007 ▶); cell refinement: *SAINT* (Bruker, 2007 ▶); data reduction: *SAINT*; program(s) used to solve structure: *SHELXS97* (Sheldrick, 2008 ▶); program(s) used to refine structure: *SHELXL97* (Sheldrick, 2008 ▶); molecular graphics: *SHELXTL* (Sheldrick, 2008 ▶); software used to prepare material for publication: *SHELXTL*.

## Supplementary Material

Crystal structure: contains datablock(s) global, I. DOI: 10.1107/S1600536813019338/lh5631sup1.cif

Structure factors: contains datablock(s) I. DOI: 10.1107/S1600536813019338/lh5631Isup2.hkl

Additional supplementary materials:  crystallographic information; 3D view; checkCIF report

## Figures and Tables

**Fig. 1.**
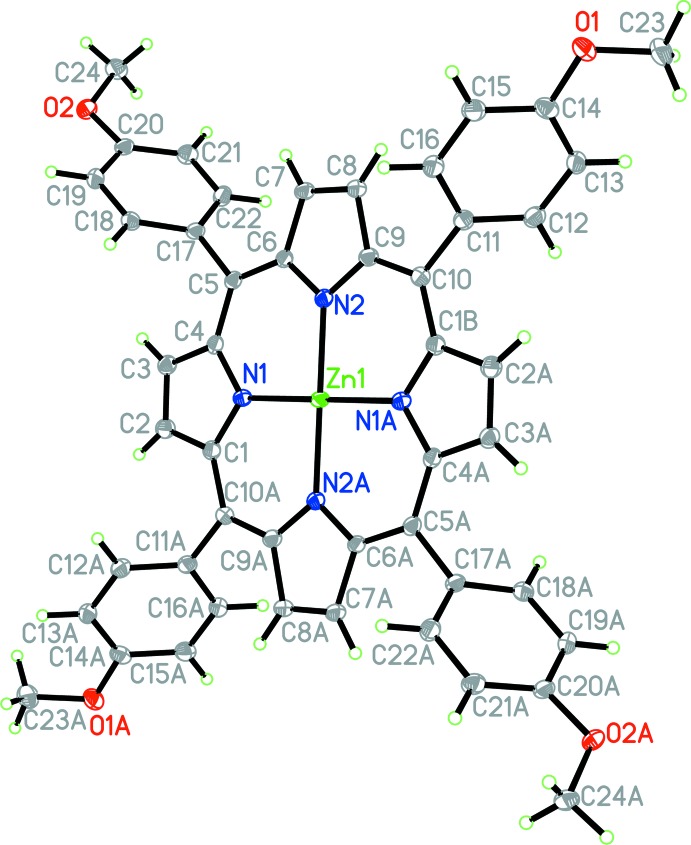
The molecular structure of the title complex with 50% probability displacement ellipsoids. The solvent molecules are not shown [symmetry code: (A) -x+1, -y+1, -z+1].

**Fig. 2.**
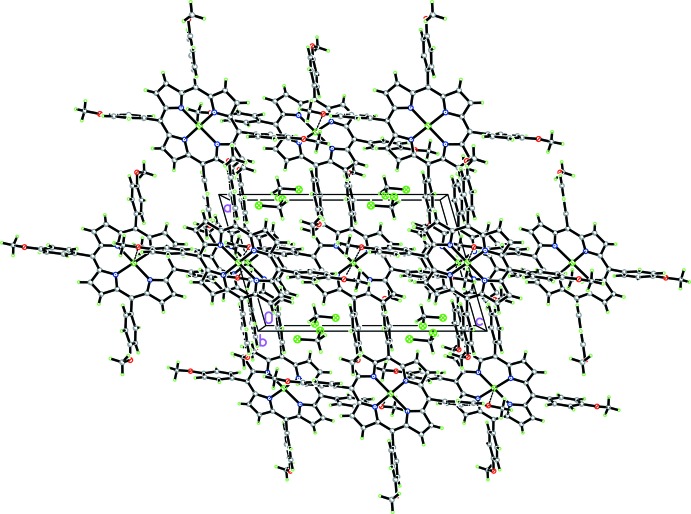
Part of the crystal structure of the title compound viewed approximately along the b axis. The short Zn···O contacts are shown as dashed lines.

## supplementary crystallographic information

### Comment

While in pursuit of understanding zinc porphyrin coordination to alkyl alcohol solvents through the central zinc atom, we sought to crystallize zinc 5,10,15,20-tetrakis(*p*-methoxyphenyl)porphyrin with octanol. The resulting deep red crystals did not contain any octanol coordination, but contained a well ordered porphyrin structure with dichloromethane solvent molecules. The crystal structure of the title compound is presented herein.

The title porphyrin (Fig. 1) has a zinc atom located on a center of inversion and hence the exact center of the mean-plane of the 24 other Non-H atoms with a r.m.s. deviation of the fitted atom = 0.0288Å. The *p*-methoxy-substituted benzene rings form dihedral angles of 59.38 (6)° (C11-C16) and 66.77 (6)° (C17-C22) with the porphyrin mean plane. The crystal packing (Fig. 2) is characterized by close contacts between the Zn^II^ ion with two symmetry related molecules (-x+1, y-1/2, -z+1/2 and x, -y+3/2, z+1/2) through the oxygen atoms of a methoxy-phenyl group (Zn···O = 2.694 (2) Å) forming a two-dimensional network parallel to (100). This distance is smaller than the sum of corresponding Van der Waals radii of atoms 2.910 Å (Bondi, 1964). There are some reports of Zn···O coordination bonds in porphyrins (Bhuyan & Sarkar, 2011; Adilov & Thalladi, 2007; Teo *et al.*, 2003). These reports indicate a bond distance shorter than what is found in the title compound.

### Experimental

The synthesis of the title Zn complex was carried out according literature procedures (Adler *et al.*, 1967). Dark red crystals were grown by liquid diffusion of 1 ml octanol into a 3 ml dichloromethane solution containing 20 mg of zinc 5,10,15,20-tetrakis- (*p*-methoxyphenyl)porphyrin.

### Refinement

All H atoms attached to C atoms were placed in idealized positions (C—H = 0.95–0.99 Å) and allowed to ride on their parent atoms. All H atoms were constrained so that *U*_iso_(H) were equal to 1.2Ueq or 1.5Ueq of their respective parent atoms.

### Crystal data

**Table:**

[Zn(C_48_H_36_N_4_O_4_)]·2CH_2_Cl_2_	*F*(000) = 996
*M**_r_* = 968.03	*D*_x_ = 1.491 Mg m^−^^3^
Monoclinic, *P*2_1_/*c*	Mo *K*α radiation, λ = 0.71073 Å
Hall symbol: -P 2ybc	Cell parameters from 8489 reflections
*a* = 11.4189 (9) Å	θ = 2.2–27.1°
*b* = 10.6877 (9) Å	µ = 0.87 mm^−^^1^
*c* = 18.3778 (15) Å	*T* = 100 K
β = 106.022 (1)°	Plate, red
*V* = 2155.7 (3) Å^3^	0.18 × 0.16 × 0.09 mm
*Z* = 2	

### Data collection

**Table:**

Bruker APEXII CCD diffractometer	4763 independent reflections
Radiation source: fine-focus sealed tube	4022 reflections with *I* > 2σ(*I*)
Graphite monochromator	*R*_int_ = 0.036
ω scans	θ_max_ = 27.1°, θ_min_ = 1.9°
Absorption correction: multi-scan (*SADABS*; Bruker, 2007)	*h* = −14→14
*T*_min_ = 0.861, *T*_max_ = 0.926	*k* = −13→13
25964 measured reflections	*l* = −23→23

### Refinement

**Table:**

Refinement on *F*^2^	Primary atom site location: structure-invariant direct methods
Least-squares matrix: full	Secondary atom site location: difference Fourier map
*R*[*F*^2^ > 2σ(*F*^2^)] = 0.039	Hydrogen site location: inferred from neighbouring sites
*wR*(*F*^2^) = 0.121	H-atom parameters constrained
*S* = 1.06	*w* = 1/[σ^2^(*F*_o_^2^) + (0.065*P*)^2^ + 2.750*P*] where *P* = (*F*_o_^2^ + 2*F*_c_^2^)/3
4763 reflections	(Δ/σ)_max_ = 0.001
288 parameters	Δρ_max_ = 0.52 e Å^−^^3^
0 restraints	Δρ_min_ = −0.78 e Å^−^^3^

### Special details

**Table:**

Geometry. All e.s.d.'s (except the e.s.d. in the dihedral angle between two l.s. planes) are estimated using the full covariance matrix. The cell e.s.d.'s are taken into account individually in the estimation of e.s.d.'s in distances, angles and torsion angles; correlations between e.s.d.'s in cell parameters are only used when they are defined by crystal symmetry. An approximate (isotropic) treatment of cell e.s.d.'s is used for estimating e.s.d.'s involving l.s. planes._iucr_refine_instructions_detailsTITL 163p21*c* in P2(1)/c *CELL* 0.71073 11.4189 10.6877 18.3778 90.000 106.022 90.000 ZERR 2.00 0.0009 0.0009 0.0015 0.000 0.001 0.000 L A T T 1 SYMM –*X*, 0.5+Y, 0.5-*Z* SFAC C H N O Cl ZN UNIT 100 80 8 8 8 2 TEMP -173.150 SIZE 0.09 0.157 0.178 acta L.S. 9 BOND $h htab FMAP 2 PLAN 20WGHT 0.065000 2.750000 FVAR 0.12081 ZN1 6 0.500000 0.500000 0.500000 10.50000 0.01678 0.01721 = 0.01254 0.00333 0.00769 0.00349 O1 4 1.215897 0.070221 0.601887 11.00000 0.01859 0.02001 = 0.02872 0.00177 0.00721 0.00549 O2 4 0.617130 0.788402 0.058282 11.00000 0.02840 0.02118 = 0.01396 0.00475 0.01136 0.00312 N1 3 0.410408 0.615123 0.412964 11.00000 0.01692 0.01511 = 0.01311 0.00039 0.00676 0.00050 N2 3 0.630415 0.466937 0.445394 11.00000 0.01750 0.01366 = 0.01246 0.00085 0.00608 0.00049 C1 1 0.302121 0.675600 0.406600 11.00000 0.01653 0.01391 = 0.01524 0.00044 0.00463 - 0.00014 C2 1 0.266170 0.741753 0.335325 11.00000 0.01740 0.01863 = 0.01566 0.00210 0.00419 0.00153 AFIX 43 H2A 2 0.194325 0.790159 0.316778 11.00000 - 1.20000 AFIX 0 C3 1 0.353693 0.721983 0.299961 11.00000 0.01963 0.01661 = 0.01433 0.00141 0.00568 - 0.00002 AFIX 43 H3A 2 0.355265 0.754260 0.252075 11.00000 - 1.20000 AFIX 0 C4 1 0.444551 0.642392 0.348779 11.00000 0.01762 0.01207 = 0.01339 0.00017 0.00512 - 0.00192 C5 1 0.548850 0.596671 0.331170 11.00000 0.01924 0.01268 = 0.01263 - 0.00106 0.00673 - 0.00246 C6 1 0.633224 0.513271 0.375828 11.00000 0.01723 0.01172 = 0.01311 - 0.00145 0.00686 - 0.00166 C7 1 0.738278 0.463970 0.356292 11.00000 0.01956 0.01661 = 0.01515 - 0.00103 0.00926 - 0.00044 AFIX 43 H7A 2 0.761143 0.480938 0.311343 11.00000 - 1.20000 AFIX 0 C8 1 0.798041 0.389162 0.413882 11.00000 0.01869 0.01599 = 0.01913 - 0.00014 0.01026 0.00066 AFIX 43 H8A 2 0.870486 0.343287 0.416895 11.00000 - 1.20000 AFIX 0 C9 1 0.730571 0.392137 0.470333 11.00000 0.01660 0.01504 = 0.01487 - 0.00104 0.00680 - 0.00111 C10 1 0.764935 0.327078 0.539542 11.00000 0.01688 0.01429 = 0.01636 - 0.00005 0.00623 0.00044 C11 1 0.882439 0.256555 0.556928 11.00000 0.01883 0.01725 = 0.01269 0.00138 0.00620 0.00189 C12 1 0.886843 0.127741 0.569024 11.00000 0.02077 0.01874 = 0.01776 0.00116 0.00814 - 0.00120 AFIX 43 H12A 2 0.813634 0.083729 0.566740 11.00000 - 1.20000 AFIX 0 C13 1 0.996564 0.062037 0.584423 11.00000 0.02345 0.01568 = 0.01776 0.00253 0.00807 0.00209 AFIX 43 H13A 2 0.997909 - 0.025748 0.592730 11.00000 - 1.20000 AFIX 0 C14 1 1.103618 0.125745 0.587519 11.00000 0.01951 0.02112 = 0.01535 - 0.00155 0.00576 0.00383 C15 1 1.100728 0.254478 0.575797 11.00000 0.01912 0.02027 = 0.02373 - 0.00157 0.00886 - 0.00081 AFIX 43 H15A 2 1.174018 0.298301 0.578027 11.00000 - 1.20000 AFIX 0 C16 1 0.991627 0.318901 0.560914 11.00000 0.02134 0.01621 = 0.01980 0.00049 0.00826 0.00002 AFIX 43 H16A 2 0.990892 0.406844 0.553285 11.00000 - 1.20000 AFIX 0 C17 1 0.571704 0.642689 0.259171 11.00000 0.01524 0.01694 = 0.01331 0.00207 0.00612 0.00109 C18 1 0.597241 0.768808 0.251103 11.00000 0.02074 0.01577 = 0.01549 - 0.00087 0.00781 0.00184 AFIX 43 H18A 2 0.603759 0.824277 0.292425 11.00000 - 1.20000 AFIX 0 C19 1 0.613282 0.814710 0.183941 11.00000 0.02334 0.01448 = 0.01985 0.00372 0.00912 0.00164 AFIX 43 H19A 2 0.631183 0.900746 0.179633 11.00000 - 1.20000 AFIX 0 C20 1 0.603100 0.734439 0.122807 11.00000 0.01661 0.02078 = 0.01560 0.00552 0.00780 0.00353 C21 1 0.582218 0.607487 0.130536 11.00000 0.02425 0.02019 = 0.01444 - 0.00264 0.00848 - 0.00086 AFIX 43 H21A 2 0.578898 0.551546 0.089844 11.00000 - 1.20000 AFIX 0 C22 1 0.566208 0.563057 0.198474 11.00000 0.02182 0.01565 = 0.01723 0.00058 0.00701 - 0.00171 AFIX 43 H22A 2 0.551213 0.476471 0.203412 11.00000 - 1.20000 AFIX 0 C23 1 1.224343 - 0.056778 0.626911 11.00000 0.02682 0.02462 = 0.02965 0.00816 0.00768 0.01065 AFIX 137 H23A 2 1.309130 - 0.084899 0.637963 11.00000 - 1.50000 H23B 2 1.172842 - 0.109520 0.587146 11.00000 - 1.50000 H23C 2 1.196853 - 0.063083 0.672799 11.00000 - 1.50000 AFIX 0 C24 1 0.631393 0.705588 0.000316 11.00000 0.03584 0.02625 = 0.01679 0.00430 0.01573 0.00822 AFIX 137 H24A 2 0.649547 0.754219 - 0.040459 11.00000 - 1.50000 H24B 2 0.555899 0.658250 - 0.019910 11.00000 - 1.50000 H24C 2 0.698519 0.647479 0.021551 11.00000 - 1.50000 AFIX 0 C L1 5 1.016160 0.452210 0.737151 11.00000 0.04632 0.03578 = 0.03553 - 0.00369 0.02023 - 0.00035 C L2 5 1.070941 0.244096 0.845764 11.00000 0.05066 0.03732 = 0.04278 0.00165 0.02344 0.00160 C1A 1 1.082372 0.301804 0.757882 11.00000 0.03466 0.04304 = 0.03536 - 0.00169 0.01402 0.00761 AFIX 23 H1AA 2 1.041196 0.242978 0.717252 11.00000 - 1.20000 H1AB 2 1.169354 0.306072 0.758752 11.00000 - 1.20000HKLF 4REM 163p21*c* in P2(1)/c REM R1 = 0.0389 for 4022 *F_o_* > 4sig(*F_o_*) and 0.0479 for all 4763 data REM 288 parameters refined using 0 restraintsENDWGHT 0.0533 3.1302 REM Highest difference peak 0.518, deepest hole -0.783, 1-sigma level 0.081 Q1 1 0.4013 0.6788 0.3211 11.00000 0.05 0.52 Q2 1 0.5621 0.6178 0.2947 11.00000 0.05 0.44 Q3 1 0.7446 0.3627 0.5013 11.00000 0.05 0.39 Q4 1 0.2799 0.7050 0.3700 11.00000 0.05 0.39 Q5 1 1.0627 0.0975 0.6026 11.00000 0.05 0.39 Q6 1 0.8242 0.2821 0.5447 11.00000 0.05 0.38 Q7 1 0.4999 0.5022 0.4666 11.00000 0.05 0.38 Q8 1 0.4430 0.6153 0.4402 11.00000 0.05 0.37 Q9 1 0.6311 0.5006 0.4190 11.00000 0.05 0.37 Q10 1 0.6781 0.4823 0.3609 11.00000 0.05 0.37 Q11 1 0.6142 0.7841 0.2163 11.00000 0.05 0.37 Q12 1 0.4338 0.5471 0.4547 11.00000 0.05 0.37 Q13 1 0.6085 0.7658 0.1538 11.00000 0.05 0.37 Q14 1 0.7640 0.3836 0.4414 11.00000 0.05 0.37 Q15 1 0.7809 0.4420 0.3877 11.00000 0.05 0.36 Q16 1 0.3624 0.6442 0.4135 11.00000 0.05 0.35 Q17 1 0.4265 0.6223 0.3814 11.00000 0.05 0.35 Q18 1 0.2755 0.6830 0.4343 11.00000 0.05 0.34 Q19 1 0.7735 0.4142 0.4510 11.00000 0.05 0.34 Q20 1 0.5817 0.5935 0.1662 11.00000 0.05 0.33
Refinement. Refinement of *F*^2^ against ALL reflections. The weighted *R*-factor *wR* and goodness of fit *S* are based on *F*^2^, conventional *R*-factors *R* are based on *F*, with *F* set to zero for negative *F*^2^. The threshold expression of *F*^2^ > σ(*F*^2^) is used only for calculating *R*-factors(gt) *etc*. and is not relevant to the choice of reflections for refinement. *R*-factors based on *F*^2^ are statistically about twice as large as those based on *F*, and *R*- factors based on ALL data will be even larger.

### Fractional atomic coordinates and isotropic or equivalent isotropic displacement parameters (Å^2^)

**Table:**

	*x*	*y*	*z*	*U*_iso_*/*U*_eq_	
Zn1	0.5000	0.5000	0.5000	0.01479 (12)	
O1	1.21590 (16)	0.07022 (16)	0.60189 (10)	0.0223 (4)	
O2	0.61713 (16)	0.78840 (16)	0.05828 (9)	0.0201 (4)	
N1	0.41041 (17)	0.61512 (18)	0.41296 (10)	0.0145 (4)	
N2	0.63042 (18)	0.46694 (18)	0.44539 (11)	0.0142 (4)	
C1	0.3021 (2)	0.6756 (2)	0.40660 (13)	0.0152 (4)	
C2	0.2662 (2)	0.7418 (2)	0.33533 (13)	0.0173 (5)	
H2A	0.1943	0.7902	0.3168	0.021*	
C3	0.3537 (2)	0.7220 (2)	0.29996 (13)	0.0167 (5)	
H3A	0.3553	0.7543	0.2521	0.020*	
C4	0.4446 (2)	0.6424 (2)	0.34878 (12)	0.0142 (4)	
C5	0.5489 (2)	0.5967 (2)	0.33117 (12)	0.0144 (4)	
C6	0.6332 (2)	0.5133 (2)	0.37583 (13)	0.0135 (4)	
C7	0.7383 (2)	0.4640 (2)	0.35629 (13)	0.0162 (4)	
H7A	0.7611	0.4809	0.3113	0.019*	
C8	0.7980 (2)	0.3892 (2)	0.41388 (13)	0.0169 (5)	
H8A	0.8705	0.3433	0.4169	0.020*	
C9	0.7306 (2)	0.3921 (2)	0.47033 (13)	0.0150 (4)	
C10	0.7649 (2)	0.3271 (2)	0.53954 (13)	0.0155 (4)	
C11	0.8824 (2)	0.2566 (2)	0.55693 (13)	0.0159 (4)	
C12	0.8868 (2)	0.1277 (2)	0.56902 (13)	0.0185 (5)	
H12A	0.8136	0.0837	0.5667	0.022*	
C13	0.9966 (2)	0.0620 (2)	0.58442 (13)	0.0185 (5)	
H13A	0.9979	−0.0257	0.5927	0.022*	
C14	1.1036 (2)	0.1257 (2)	0.58752 (13)	0.0185 (5)	
C15	1.1007 (2)	0.2545 (2)	0.57580 (14)	0.0205 (5)	
H15A	1.1740	0.2983	0.5780	0.025*	
C16	0.9916 (2)	0.3189 (2)	0.56091 (14)	0.0186 (5)	
H16A	0.9909	0.4068	0.5533	0.022*	
C17	0.5717 (2)	0.6427 (2)	0.25917 (13)	0.0147 (4)	
C18	0.5972 (2)	0.7688 (2)	0.25110 (13)	0.0168 (5)	
H18A	0.6038	0.8243	0.2924	0.020*	
C19	0.6133 (2)	0.8147 (2)	0.18394 (14)	0.0186 (5)	
H19A	0.6312	0.9007	0.1796	0.022*	
C20	0.6031 (2)	0.7344 (2)	0.12281 (13)	0.0170 (5)	
C21	0.5822 (2)	0.6075 (2)	0.13054 (13)	0.0190 (5)	
H21A	0.5789	0.5515	0.0898	0.023*	
C22	0.5662 (2)	0.5631 (2)	0.19847 (13)	0.0179 (5)	
H22A	0.5512	0.4765	0.2034	0.021*	
C23	1.2243 (3)	−0.0568 (3)	0.62691 (16)	0.0270 (6)	
H23A	1.3091	−0.0849	0.6380	0.041*	
H23B	1.1728	−0.1095	0.5871	0.041*	
H23C	1.1969	−0.0631	0.6728	0.041*	
C24	0.6314 (3)	0.7056 (3)	0.00032 (14)	0.0246 (5)	
H24A	0.6495	0.7542	−0.0405	0.037*	
H24B	0.5559	0.6582	−0.0199	0.037*	
H24C	0.6985	0.6475	0.0216	0.037*	
Cl1	1.01616 (7)	0.45221 (7)	0.73715 (4)	0.03743 (19)	
Cl2	1.07094 (8)	0.24410 (8)	0.84576 (5)	0.0415 (2)	
C1A	1.0824 (3)	0.3018 (3)	0.75788 (18)	0.0368 (7)	
H1AA	1.0412	0.2430	0.7173	0.044*	
H1AB	1.1694	0.3061	0.7588	0.044*	

### Atomic displacement parameters (Å^2^)

**Table:**

	*U*^11^	*U*^22^	*U*^33^	*U*^12^	*U*^13^	*U*^23^
Zn1	0.0168 (2)	0.0172 (2)	0.01254 (19)	0.00349 (14)	0.00769 (14)	0.00333 (14)
O1	0.0186 (8)	0.0200 (9)	0.0287 (10)	0.0055 (7)	0.0072 (7)	0.0018 (7)
O2	0.0284 (9)	0.0212 (9)	0.0140 (8)	0.0031 (7)	0.0114 (7)	0.0047 (7)
N1	0.0169 (9)	0.0151 (9)	0.0131 (9)	0.0005 (7)	0.0068 (7)	0.0004 (7)
N2	0.0175 (9)	0.0137 (9)	0.0125 (9)	0.0005 (7)	0.0061 (7)	0.0008 (7)
C1	0.0165 (11)	0.0139 (10)	0.0152 (11)	−0.0001 (8)	0.0046 (9)	0.0004 (8)
C2	0.0174 (11)	0.0186 (11)	0.0157 (11)	0.0015 (9)	0.0042 (9)	0.0021 (9)
C3	0.0196 (11)	0.0166 (11)	0.0143 (11)	0.0000 (9)	0.0057 (9)	0.0014 (9)
C4	0.0176 (11)	0.0121 (10)	0.0134 (10)	−0.0019 (8)	0.0051 (8)	0.0002 (8)
C5	0.0192 (11)	0.0127 (10)	0.0126 (10)	−0.0025 (8)	0.0067 (8)	−0.0011 (8)
C6	0.0172 (11)	0.0117 (10)	0.0131 (10)	−0.0017 (8)	0.0069 (8)	−0.0014 (8)
C7	0.0196 (11)	0.0166 (11)	0.0151 (11)	−0.0004 (9)	0.0093 (9)	−0.0010 (8)
C8	0.0187 (11)	0.0160 (11)	0.0191 (11)	0.0007 (9)	0.0103 (9)	−0.0001 (9)
C9	0.0166 (10)	0.0150 (10)	0.0149 (10)	−0.0011 (8)	0.0068 (8)	−0.0010 (9)
C10	0.0169 (11)	0.0143 (10)	0.0164 (11)	0.0004 (8)	0.0062 (9)	−0.0001 (8)
C11	0.0188 (11)	0.0173 (11)	0.0127 (10)	0.0019 (9)	0.0062 (9)	0.0014 (8)
C12	0.0208 (12)	0.0187 (12)	0.0178 (11)	−0.0012 (9)	0.0081 (9)	0.0012 (9)
C13	0.0234 (12)	0.0157 (11)	0.0178 (11)	0.0021 (9)	0.0081 (9)	0.0025 (9)
C14	0.0195 (11)	0.0211 (12)	0.0153 (11)	0.0038 (9)	0.0058 (9)	−0.0016 (9)
C15	0.0191 (11)	0.0203 (12)	0.0237 (12)	−0.0008 (9)	0.0089 (10)	−0.0016 (10)
C16	0.0213 (12)	0.0162 (11)	0.0198 (12)	0.0000 (9)	0.0083 (9)	0.0005 (9)
C17	0.0152 (10)	0.0169 (11)	0.0133 (10)	0.0011 (8)	0.0061 (8)	0.0021 (8)
C18	0.0207 (11)	0.0158 (11)	0.0155 (11)	0.0018 (9)	0.0078 (9)	−0.0009 (9)
C19	0.0233 (12)	0.0145 (11)	0.0199 (12)	0.0016 (9)	0.0091 (9)	0.0037 (9)
C20	0.0166 (11)	0.0208 (11)	0.0156 (11)	0.0035 (9)	0.0078 (9)	0.0055 (9)
C21	0.0243 (12)	0.0202 (12)	0.0144 (11)	−0.0009 (9)	0.0085 (9)	−0.0026 (9)
C22	0.0218 (12)	0.0157 (11)	0.0172 (11)	−0.0017 (9)	0.0070 (9)	0.0006 (9)
C23	0.0268 (13)	0.0246 (13)	0.0296 (14)	0.0106 (11)	0.0077 (11)	0.0082 (11)
C24	0.0358 (14)	0.0263 (13)	0.0168 (12)	0.0082 (11)	0.0157 (11)	0.0043 (10)
Cl1	0.0463 (4)	0.0358 (4)	0.0355 (4)	−0.0003 (3)	0.0202 (3)	−0.0037 (3)
Cl2	0.0507 (5)	0.0373 (4)	0.0428 (4)	0.0016 (3)	0.0234 (4)	0.0017 (3)
C1A	0.0347 (16)	0.0430 (18)	0.0354 (16)	0.0076 (13)	0.0140 (13)	−0.0017 (14)

### Geometric parameters (Å, º)

**Table:**

Zn1—N2^i^	2.0432 (19)	C11—C16	1.398 (3)
Zn1—N2	2.0432 (19)	C12—C13	1.395 (3)
Zn1—N1^i^	2.0532 (19)	C12—H12A	0.9500
Zn1—N1	2.0532 (19)	C13—C14	1.387 (3)
O1—C14	1.371 (3)	C13—H13A	0.9500
O1—C23	1.428 (3)	C14—C15	1.392 (3)
O2—C20	1.367 (3)	C15—C16	1.383 (3)
O2—C24	1.428 (3)	C15—H15A	0.9500
N1—C1	1.371 (3)	C16—H16A	0.9500
N1—C4	1.372 (3)	C17—C22	1.391 (3)
N2—C9	1.367 (3)	C17—C18	1.396 (3)
N2—C6	1.380 (3)	C18—C19	1.387 (3)
C1—C10^i^	1.409 (3)	C18—H18A	0.9500
C1—C2	1.445 (3)	C19—C20	1.393 (3)
C2—C3	1.351 (3)	C19—H19A	0.9500
C2—H2A	0.9500	C20—C21	1.392 (3)
C3—C4	1.446 (3)	C21—C22	1.394 (3)
C3—H3A	0.9500	C21—H21A	0.9500
C4—C5	1.405 (3)	C22—H22A	0.9500
C5—C6	1.400 (3)	C23—H23A	0.9800
C5—C17	1.500 (3)	C23—H23B	0.9800
C6—C7	1.443 (3)	C23—H23C	0.9800
C7—C8	1.351 (3)	C24—H24A	0.9800
C7—H7A	0.9500	C24—H24B	0.9800
C8—C9	1.453 (3)	C24—H24C	0.9800
C8—H8A	0.9500	Cl1—C1A	1.773 (3)
C9—C10	1.407 (3)	Cl2—C1A	1.767 (3)
C10—C1^i^	1.409 (3)	C1A—H1AA	0.9900
C10—C11	1.495 (3)	C1A—H1AB	0.9900
C11—C12	1.393 (3)		
			
N2^i^—Zn1—N2	180.00 (11)	C13—C12—H12A	119.3
N2^i^—Zn1—N1^i^	89.71 (8)	C14—C13—C12	119.6 (2)
N2—Zn1—N1^i^	90.29 (8)	C14—C13—H13A	120.2
N2^i^—Zn1—N1	90.29 (8)	C12—C13—H13A	120.2
N2—Zn1—N1	89.71 (8)	O1—C14—C13	124.2 (2)
N1^i^—Zn1—N1	180.0	O1—C14—C15	116.0 (2)
C14—O1—C23	116.75 (19)	C13—C14—C15	119.7 (2)
C20—O2—C24	116.76 (19)	C16—C15—C14	120.3 (2)
C1—N1—C4	106.86 (18)	C16—C15—H15A	119.9
C1—N1—Zn1	126.34 (15)	C14—C15—H15A	119.9
C4—N1—Zn1	126.75 (15)	C15—C16—C11	121.0 (2)
C9—N2—C6	106.82 (18)	C15—C16—H16A	119.5
C9—N2—Zn1	126.33 (15)	C11—C16—H16A	119.5
C6—N2—Zn1	126.84 (15)	C22—C17—C18	118.1 (2)
N1—C1—C10^i^	125.6 (2)	C22—C17—C5	121.8 (2)
N1—C1—C2	109.38 (19)	C18—C17—C5	120.2 (2)
C10^i^—C1—C2	125.0 (2)	C19—C18—C17	121.3 (2)
C3—C2—C1	107.3 (2)	C19—C18—H18A	119.4
C3—C2—H2A	126.4	C17—C18—H18A	119.4
C1—C2—H2A	126.4	C18—C19—C20	119.9 (2)
C2—C3—C4	107.0 (2)	C18—C19—H19A	120.1
C2—C3—H3A	126.5	C20—C19—H19A	120.1
C4—C3—H3A	126.5	O2—C20—C21	124.3 (2)
N1—C4—C5	125.7 (2)	O2—C20—C19	115.9 (2)
N1—C4—C3	109.45 (19)	C21—C20—C19	119.8 (2)
C5—C4—C3	124.8 (2)	C20—C21—C22	119.5 (2)
C6—C5—C4	125.2 (2)	C20—C21—H21A	120.3
C6—C5—C17	117.8 (2)	C22—C21—H21A	120.3
C4—C5—C17	117.0 (2)	C17—C22—C21	121.5 (2)
N2—C6—C5	125.7 (2)	C17—C22—H22A	119.3
N2—C6—C7	109.45 (19)	C21—C22—H22A	119.3
C5—C6—C7	124.8 (2)	O1—C23—H23A	109.5
C8—C7—C6	107.2 (2)	O1—C23—H23B	109.5
C8—C7—H7A	126.4	H23A—C23—H23B	109.5
C6—C7—H7A	126.4	O1—C23—H23C	109.5
C7—C8—C9	107.1 (2)	H23A—C23—H23C	109.5
C7—C8—H8A	126.5	H23B—C23—H23C	109.5
C9—C8—H8A	126.5	O2—C24—H24A	109.5
N2—C9—C10	126.2 (2)	O2—C24—H24B	109.5
N2—C9—C8	109.44 (19)	H24A—C24—H24B	109.5
C10—C9—C8	124.3 (2)	O2—C24—H24C	109.5
C9—C10—C1^i^	125.1 (2)	H24A—C24—H24C	109.5
C9—C10—C11	116.9 (2)	H24B—C24—H24C	109.5
C1^i^—C10—C11	118.0 (2)	Cl2—C1A—Cl1	112.23 (17)
C12—C11—C16	118.1 (2)	Cl2—C1A—H1AA	109.2
C12—C11—C10	121.5 (2)	Cl1—C1A—H1AA	109.2
C16—C11—C10	120.4 (2)	Cl2—C1A—H1AB	109.2
C11—C12—C13	121.4 (2)	Cl1—C1A—H1AB	109.2
C11—C12—H12A	119.3	H1AA—C1A—H1AB	107.9

Symmetry code: (i) −*x*+1, −*y*+1, −*z*+1.
